# Electronic Screen Use and Sleep Duration and Timing in Adults

**DOI:** 10.1001/jamanetworkopen.2025.2493

**Published:** 2025-03-27

**Authors:** Charlie Zhong, Matthew Masters, Sidney M. Donzella, W. Ryan Diver, Alpa V. Patel

**Affiliations:** 1 Department of Population Science, American Cancer Society, Atlanta, Georgia; 2 Department of Epidemiology, University of Washington, Seattle

## Abstract

**Importance:**

Electronic screen use before bed may disrupt circadian rhythms, leading to poorer sleep.

**Objective:**

To investigate the association between screen use before bed and sleep outcomes among adults.

**Design, Setting, and Participants:**

This cross-sectional study included participants in the American Cancer Society Cancer Prevention Study–3, a prospective cohort of men and women from 35 US states and Puerto Rico, who responded to a 2018 survey. Analyses were conducted from February 3, 2023, to January 10, 2025.

**Exposures:**

Self-reported electronic screen use in the hour prior to bed.

**Main Outcomes and Measures:**

Self-reported sleep-wake times (used to calculate duration), sleep quality, and chronotype. Mean differences for sleep duration and bedtimes, and prevalence ratios for sleep quality were calculated by screen use using multivariable linear and Poisson regression, respectively. Whether these associations differed by chronotype was also examined.

**Results:**

Of the 122 058 participants (97 658 women [80.0%]; median [IQR] age, 56 [47-62] years; range, 27-85 years), 70 638 (57.9%) reported a morning chronotype. Daily screen use before bed was reported by 50 289 participants (41.2%), whereas 21 275 (17.4%) reported no screen use. Compared with no screen use, daily screen use prior to bed was associated with a 33% higher prevalence of poor sleep quality (prevalence ratio, 1.33; 95% CI, 1.27-1.39) and 7.64 fewer minutes of sleep on workdays (95% CI, 6.65-8.63 minutes). The association was more pronounced among participants with evening chronotypes (8.36 minutes; 95% CI, 4.94-11.78 minutes) vs those with morning chronotypes (5.64 minutes; 95% CI, 3.98-7.29 minutes). Daily screen use was associated with 5.04 fewer minutes of sleep (95% CI, 4.03-6.05 minutes) on nonworkdays. Daily screen users with morning chronotype went to bed 9.33 minutes later (95% CI, 7.61-11.06 minutes), and those with evening chronotypes went to bed 15.62 minutes later (95% CI, 11.93-19.31 minutes) on workdays. Results were similar for nonworkdays.

**Conclusions and Relevance:**

Daily screen use was associated with later bedtimes and approximately 50 minutes less sleep each week. Associations were greater among those with evening chronotypes, who are at risk for poor sleep due to social jetlag (ie, misalignment between circadian rhythms and social commitments). These findings confirm disruptions to sleep from electronic screens are not limited to children and adolescents. Further work is needed to understand the best mechanisms for intervention.

## Key Points

**Question:**

Is use of an electronic screen before bed associated with sleep outcomes in adults?

**Findings:**

In this cross-sectional analysis of 122 058 participants in the American Cancer Society Cancer Prevention Study–3, screen use was associated with decreased sleep duration and worse self-reported sleep quality. These associations were more pronounced in participants with a later chronotype.

**Meaning:**

These findings expand on the literature surrounding screen use and poorer sleep outcomes by confirming the associations among an adult population, especially among those with a later chronotype.

## Teaser

This cross-sectional study examines the association between screen use before bed and sleep outcomes among adults.

## Introduction

Adequate and high-quality sleep is essential for good health. However, average sleep duration and quality has declined the past several decades, with approximately one-third of adults not meeting the recommended guidelines of 7 to 9 hours of sleep per night.^1^ Light is one of the main environmental factors affecting the sleep-wake cycle. Photosensitive cells in our retina respond to light, and the absence of light at nighttime leads to the increased secretion of melatonin, which, in turn, contributes to the feeling of sleepiness.^2,3^ Exposure to bright lights before sleep can delay the release of melatonin, therefore increasing sleep latency and disrupting circadian rhythm.^4^ Our light environment has undergone drastic changes in the last several decades. Increasing urbanization has led to annual increase in artificial light at night (ALAN) in our environment,^5^ and several reviews^2,6,7,8^ have been published on the associations between ALAN and adverse health outcomes, including disruptions to sleep.

Of more recent concern is the direct exposure to ALAN though electronic screen use. As of 2022, the prevalence of smartphone ownership in the US has reached 85%, up from just 35% a decade ago.^9^ These devices commonly use light-emitting diodes as the main source of illumination. The intensity of the light emitted in these devices generally peak in the 450 nm range, corresponding to the blue spectrum of light,^10^ which may be especially disruptive to human sleep.^11,12^ A majority of mobile device owners report looking at their screen prior to sleep, which may be one of the contributing factors to the recent decline in sleep duration and quality.^13,14^

Much of the research focused on electronic screen use and sleep disruption has been conducted in adolescent and young adult populations, in part owing to their earlier adoption of such technologies, as well as the ease of obtaining participants (eg, in a university setting).^15,16,17,18^ However, behavioral (eg, office workers spend more time indoors compared with adolescents) and sleep patterns among individuals in this age range differ from those of adults. Chronotype, the body’s natural internal clock, is latest around age 18 years and gradually shifts toward an earlier chronotype as we age.^3,19^ Adolescents may also be less sensitive to light-induced sleep disruption. In this study, we cross-sectionally evaluated electronics screen use prior to sleep and sleep outcomes in US adults.

## Methods

### Study Population

This cross-sectional analysis was conducted within the American Cancer Society Cancer Prevention Study-3 (CPS-3) cohort. CPS-3 is a prospective cohort of men and women enrolled in 2006 to 2013 from 35 US states and Puerto Rico.^20^ CPS-3 was approved by the institutional review board of Emory University. All CPS-3 participants provided written consent when they completed the on-site survey at enrollment, with trained volunteers serving as the witness. This study followed the Strengthening the Reporting of Observational Studies in Epidemiology (STROBE) reporting guidelines.

At baseline, participants self-reported demographics, including age, sex, race and ethnicity, and education. Choices provided for race and ethnicity included American Indian or Alaska Native, Asian, Black, Hispanic, Native Hawaiian or Pacific Islander, White, and other (with a write-in text box). Participants were able to select more than 1 choice. Race and ethnicity were included in this study to better describe the study population. The CPS-3 continues to regularly follow up with its participants, including triennial questionnaires. We evaluated electronic screen use and sleep using responses collected among participants who responded to the 2018 triennial questionnaire.

### Electronic Screen Use

To evaluate electronic screen use prior to sleep, participants were asked to self-report, “In a typical week, how often do you watch or read an electronic screen not including TV (i.e. smartphone, laptop, tablet) in the hour before sleeping?” on the 2018 triennial follow-up questionnaire. Additional questions related to the bedroom light environment included use of a sleep eye mask and brightness of the bedroom while sleeping (too dark to see your hand, light enough to see your hand in front of you but not to see across the room, light enough to see across the room but not read, or light enough to read).

### Sleep

Various sleep outcomes were self-reported on the 2018 questionnaire. Participants reported the average clock time in the past year at which they typically tried to fall asleep and wake up, separately for workdays and nonworkdays. Participants were also asked to indicate how many days corresponded to workdays and nonworkdays (eFigure 1 in Supplement 1). We considered a participant to have an active work status if they reported having a workday during the week, which included participants working full-time, part-time, volunteering, or engaged in any other activity for which they would have reported a different sleep pattern from nonworkdays. If they reported that they did not work during the past year, they only reported nonworkday sleep and wake times. A few participants also reported working every day of the week, in which case they only reported workday sleep and wake times. We calculated sleep duration as the difference, in minutes, between reported wake time and sleep time and have validated this against actigraphy and sleep diaries within the CPS-3.^21^ Overall sleep quality was assessed with the single-item quality question from the Pittsburgh Sleep Quality Index,^22^ with the choices of very poor, fairly poor, fairly well, and very well. The Pittsburgh Sleep Quality Index is the most widely used sleep assessment tool and has demonstrated good validity overall and for the single-item sleep quality subscale question.^23^ To our knowledge, an individual evaluation of this question on its own has not been undertaken. Our assessment of chronotype was based on the final item from the Horne-Östberg Morningness-Eveningness Questionnaire,^24^ if participants consider themselves definitely a morning type, more of a morning than an evening type, more of an evening than a morning type, definitely an evening type, or none. This single item has shown strong correlation with the full Horne-Östberg Morningness-Eveningness Questionnaire.^25,26^

### Statistical Analysis

To evaluate the effect of electronic screen use on sleep, we calculated prevalence ratios (PRs) and 95% CIs for sleep quality (very well and fairly well vs very poor and fairly poor) using a Poisson regression with robust SEs,^27,28^ and mean (95% CI) change in minutes of overnight sleep for workday and nonworkday sleep duration and bedtimes with linear regression. Our base model adjusted for age and sex. In fully adjusted models, we adjusted for age, sex, race, ethnicity, work status, education, bedroom brightness, and sleep mask use. In sleep quality and nonworkday models, we further adjusted for work status. We further stratified by chronotype (with none as the reference) to assess the influence an individual’s internal circadian clock has on the association between electronic screen use and sleep. Finally, we computed E-values for all our tests. An E-value is the minimum association needed for an unmeasured confounder to explain away an estimated effect.^29^ All analyses were performed using R statistical software version 4.2.3 (R Project for Statistical Computing) between February 3, 2023, and January 10, 2025.

## Results

 Among the 164 826 participants who responded to the 2018 questionnaire, we excluded 27 627 participants who reported an invalid number of work and nonworkdays in a week, because we would be unable to calculate a weekly average duration, 4891 participants who reported extreme sleep durations (<3 hours or >14 hours), 2363 participants who were missing responses related to electronic screen use and sleep, and 7887 who reported shiftwork, for a total of 122 058 participants in this analysis (eFigure 2 in Supplement 1). The median (IQR) age among the participants who reported device use and sleep on the 2018 follow-up questionnaire was 56 (47-62) years (range, 27-85 years). Most participants were White (108 888 participants [89.2%]), were women (97 658 participants [80.0%]), and had completed a college degree (70 823 participants [58.0%]), and closely reflected the distributions seen in the cohort at baseline.^20^ In addition, 2638 participants (2.2%) were Black, 6201 (5.1%) were Hispanic, and 4331 (3.5%) were of another race or ethnicity (ie, American Indian or Alaska Native, Asian, Native Hawaiian or Pacific Islander, and write-in responses). Participants with morning chronotypes (70 638 participants [57.9%]) were more common than those with evening chronotypes (43 880 participants [35.9%]). Few participants reported use of an eye mask when sleeping (4424 participants [3.6%]), and most reported their bedrooms to be dark enough that they could not see across the room (99 066 participants [81.1%]).

A large portion of our participants reported electronic screen use prior to sleep every night of the week (50 289 participants [41.2%]) (Table 1). In contrast, 21 275 participants (17.4%) reported no screen use prior to sleep, and the rest reported occasional use. Those reporting no use were older (median [IQR] age, 60 [53-65] vs 56 [47-62] years) and were more likely to be male (4879 male participants [22.9%] vs 24 400 male participants [20.0%]) compared with the overall population. Compared with no screen use, those who reported daily screen use were more likely to report less than the recommend 7 to 9 hours of sleep on both workdays (8391 participants [19.1%] vs 2544 participants [15.0%]) and nonworkdays (2549 participants [5.1%] vs 899 participants [4.2%]). A total of 17 349 participants reported only nonworkday sleep times, and 258 reported working every day of the week.

**Table 1.** Demographic Characteristics of Cancer Prevention Study–3 Participants in 2018

Characteristic	Participants, No. (%), by frequency of screen use in the hour before sleep				
	0 d/wk (n = 21 275)	1-3 d/wk (n = 20 918)	4-6 d/wk (n = 29 576)	7 d/wk (n = 50 289)	Total (N = 122 058)
Age, median (IQR), y	60 (53-65)	56 (49-62)	55 (46-61)	54 (45-61)	56 (47-62)
Sex					
Male	4879 (22.9)	4220 (20.2)	5992 (20.3)	9309 (18.5)	24 400 (20.0)
Female	16 396 (77.1)	16 698 (79.8)	23 584 (79.7)	40 980 (81.5)	97 658 (80.0)
Ever own cellular telephone					
No	175 (0.8)	43 (0.2)	73 (0.2)	109 (0.2)	400 (0.3)
Yes, in past	170 (0.8)	102 (0.5)	110 (0.4)	173 (0.3)	555 (0.5)
Yes, currently	20 731 (97.4)	20 611 (98.5)	29 150 (98.6)	49 512 (98.5)	120 004 (98.3)
Missing	199 (0.9)	162 (0.8)	243 (0.8)	495 (1.0)	1099 (0.9)
Race and ethnicity					
Black	395 (1.9)	470 (2.2)	608 (2.1)	1165 (2.3)	2638 (2.2)
Hispanic	866 (4.1)	1048 (5.0)	1522 (5.1)	2765 (5.5)	6201 (5.1)
White	19 398 (91.2)	18 740 (89.6)	26 481 (89.5)	44 269 (88.0)	108 888 (89.2)
Other^a^	616 (2.9)	660 (3.2)	965 (3.3)	2090 (4.2)	4331 (3.5)
Education					
High school or less	1162 (5.5)	857 (4.1)	1135 (3.8)	2157 (4.3)	5311 (4.4)
Some college or 2-y degree	4354 (20.5)	3807 (18.2)	5286 (17.9)	10 346 (20.6)	23 793 (19.5)
4-y College degree	5881 (27.6)	6376 (30.5)	9134 (30.9)	15 214 (30.3)	36 605 (30.0)
Graduate degree	5496 (25.8)	6042 (28.9)	8794 (29.7)	13 886 (27.6)	34 218 (28.0)
Missing	4382 (20.6)	3836 (18.3)	5227 (17.7)	8686 (17.3)	22 131 (18.1)
Workday sleep duration, median (IQR), min	465 (420-480)	450 (420-480)	450 (420-480)	450 (420-480)	450 (420-480)
Nonworkers, No.^b^	4348	3062	3627	6312	17 349
Workday sleep duration categorized					
<7 h	2544 (15.0)	2729 (15.3)	4457 (17.2)	8391 (19.1)	18 121 (17.3)
7-9 h	13 849 (81.8)	14 700 (82.3)	20 926 (80.6)	34 375 (78.2)	83 850 (80.1)
>9 h	534 (3.2)	427 (2.4)	566 (2.2)	1211 (2.7)	2738 (2.6)
Nonworkday sleep duration, median (IQR), min	498 (480-540)	480 (480-540)	490 (480-540)	490 (480-540)	495 (480-540)
Worked 7 d, No.^c^	54	30	42	132	258
Nonworkday sleep duration categorized					
<7 h	899 (4.2)	776 (3.7)	1130 (3.8)	2549 (5.1)	5354 (4.4)
7-9 h	17 081 (80.5)	17 177 (82.2)	24 368 (82.5)	39 766 (79.3)	98 392 (80.8)
>9 h	3241 (15.3)	2935 (14.1)	4036 (13.7)	7842 (15.6)	18 054 (14.8)
Sleep quality					
Very poor	461 (2.2)	428 (2.0)	555 (1.9)	1450 (2.9)	2894 (2.4)
Fairly poor	3217 (15.1)	3497 (16.7)	5099 (17.2)	9641 (19.2)	21 454 (17.6)
Fairly well	13 024 (61.2)	13 199 (63.1)	18 899 (63.9)	30 844 (61.3)	75 966 (62.2)
Very well	4573 (21.5)	3794 (18.1)	5023 (17.0)	8354 (16.6)	21 744 (17.8)
Reported chronotype					
Morning	8138 (38.3)	6497 (31.1)	8109 (27.4)	12 997 (25.8)	35 741 (29.3)
More morning than evening	6227 (29.3)	6498 (31.1)	8737 (29.5)	13 435 (26.7)	34 897 (28.6)
More evening than morning	3712 (17.4)	4455 (21.3)	7047 (23.8)	11 805 (23.5)	27 019 (22.1)
Evening	1848 (8.7)	2194 (10.5)	3911 (13.2)	8908 (17.7)	16 861 (13.8)
None	1308 (6.2)	1235 (5.9)	1698 (5.7)	3016 (6.0)	7257 (5.9)
Missing	42 (0.2)	39 (0.2)	74 (0.3)	128 (0.3)	283 (0.2)
Sleep eye mask use					
No	20 552 (96.6)	20 113 (96.2)	28 494 (96.3)	48 475 (96.4)	117 634 (96.4)
Yes	723 (3.4)	805 (3.8)	1082 (3.7)	1814 (3.6)	4424 (3.6)
Bedroom light					
Too dark to see hand	7399 (34.8)	6849 (32.7)	9761 (33.0)	16 203 (32.2)	40 212 (32.9)
See hand, not across room	10 015 (47.1)	10 361 (49.5)	14 668 (49.6)	23 810 (47.3)	58 854 (48.2)
See across room, not read	3618 (17.0)	3435 (16.4)	4698 (15.9)	9370 (18.6)	21 121 (17.3)
Enough to read	243 (1.1)	273 (1.3)	449 (1.5)	906 (1.8)	1871 (1.5)

^a^ Other includes self-reported American Indian or Alaska Native, Asian, Native Hawaiian or Pacific Islander, and write-in responses.

^b^ Participants who indicated 0 workdays only reported nonworkday sleep and wake times.

^c^ Participants who reported working every day of the week only reported workday sleep and wake times.

In age-adjusted and sex-adjusted models (Table 2), compared with no screen use in the hour before bed, those who reported daily use slept a mean of 7.78 fewer minutes (95% CI, 6.79-8.77 minutes; E-value, 1.53) and reported 19.01 minutes (95% CI, 17.90-20.13 minutes; E-value, 1.97) later bedtimes on workdays. On nonworkdays, daily screen users reported 5.20 fewer minutes of sleep (95% CI, 4.19-6.21 minutes; E-value, 1.37) and 19.87 minutes later bedtimes (95% CI, 18.74-20.99 minutes; E-value, 1.92). Daily screen use was associated with 26% increased prevalence (PR, 1.26; 95% CI, 1.21-1.30; E-value, 1.49) (Table 3) of reporting poor sleep quality. The E-values ranged from 1.24 for sleep quality to 1.96 for workday bedtimes, suggesting, at minimum, that an unmeasured confounder would need to exert an effect size of 1.24 to negate our results.

**Table 2.** Associations Between Electronic Screen Use Before Bed and Sleep Duration and Timing in the Cancer Prevention Study–3

Variable	Sleep duration				Bedtimes			
	Model 1		Model 2		Model 1		Model 2	
	Change, mean (95% CI), min^a^	E-value	Change, mean (95% CI), min^b^	E-value	Change, mean (95% CI), min^a^	E-value	Change, mean (95% CI), min^b^	e-Value
Workday (n = 104 709)								
No screen use	0 [Reference]	NA	0 [Reference]	NA	0 [Reference]	NA	0 [Reference]	NA
Use 1-3 d/wk	−3.41 (−4.57 to −2.25)	1.31	−3.40 (−4.55 to −2.24)	1.30	7.25 (5.95 to 8.56)	1.46	6.87 (5.57 to 8.17)	1.45
Use 4-6 d/wk	−6.35 (−7.43 to −5.28)	1.46	−6.35 (−7.42 to −5.28)	1.46	13.74 (12.53 to 14.95)	1.74	13.38 (12.18 to 14.59)	1.73
Daily use	−7.78 (−8.77 to −6.79)	1.53	−7.64 (−8.63 to −6.65)	1.53	19.01 (17.90 to 20.13)	1.97	18.82 (17.70 to 19.93)	1.96
Nonworkday (n = 121 800)								
No screen use	0 [Reference]		0 [Reference]	NA	0 [Reference]	NA	0 [Reference]	NA
Use 1-3 d/wk	−4.26 (−5.45 to −3.07)	1.32	−4.08 (−5.26 to −2.89)	1.32	8.77 (7.45 to 10.09)	1.49	8.56 (7.25 to 9.88)	1.48
Use 4-6 d/wk	−5.14 (−6.25 to −4.04)	1.37	−4.95 (−6.06 to −3.85)	1.36	15.05 (13.82 to 16.27)	1.73	14.93 (13.71 to 16.15)	1.73
Daily use	−5.20 (−6.21 to −4.19)	1.37	−5.04 (−6.05 to −4.03)	1.36	19.87 (18.74 to 20.99)	1.92	19.69 (18.56 to 20.81)	1.91

Abbreviation: NA, not applicable.

^a^ Adjusted for age and sex.

^b^ Adjusted for age, sex, race, ethnicity, education, bedroom light environment, and sleep mask use; nonworkday models additionally were adjusted for work status.

**Table 3.** Associations Between Electronic Screen Use Before Bed and Sleep Quality in the Cancer Prevention Study–3

Variable	Model 1 (n = 122 058)^a^		Model 2 (n = 122 058)^b^	
	PR (95% CI)	E-value	PR (95% CI)	E-value
No screen use	1 [Reference]	NA	1 [Reference]	NA
Use 1-3 d/wk	1.08 (1.03-1.12)	1.24	1.11 (1.05-1.16)	1.29
Use 4-6 d/wk	1.09 (1.05-1.14)	1.26	1.14 (1.08-1.19)	1.34
Daily use	1.26 (1.21-1.30)	1.49	1.33 (1.27-1.39)	1.57

Abbreviations: NA, not applicable; PR, prevalence ratio.

^a^ Adjusted for age and sex.

^b^ Adjusted for age, sex, race, ethnicity, education, bedroom light environment, and sleep mask use.

After adjusting for race, ethnicity, education, work status, bedroom light environment, and mask use during sleep, daily screen use was associated with 7.64 fewer minutes of sleep (95% CI, 6.65-8.63 minutes) (Table 2) on workdays and 5.04 fewer minutes of sleep on nonworkdays (95% CI, 4.03-6.05 minutes). In fully adjusted models, daily screen use was associated with going to bed 18.82 minutes later (95% CI, 17.70-19.93 minutes) on workdays and 19.69 minutes later (95% CI, 18.56-20.81 minutes) on nonworkdays. The prevalence of poor sleep quality was 33% (PR, 1.33; 95% CI, 1.27-1.39) higher among daily users compared with those reporting no screen use.

Participants reporting later chronotypes had later bedtimes (eTable in Supplement 1). Compared with participants reporting neither morning nor evening chronotype, those reporting morning chronotype had a bedtime a mean of 34 minutes earlier on workdays and nonworkdays, whereas those with evening chronotypes reported later bedtimes on workdays by 44 minutes and on nonworkdays by 53 minutes. When stratified by chronotype (Table 4), the association between screen use and sleep duration and timing was more pronounced among evening chronotypes than morning. On average, participants with evening chronotypes with daily screen use reported bedtimes that were 15.62 minutes later (95% CI, 11.93-19.31 minutes) with a corresponding 8.36 fewer minutes of sleep duration (95% CI, 4.94-11.78 minutes) on workdays, compared with participants with morning chronotypes with daily screen use reporting only 9.33 minutes later bedtimes (95% CI, 7.61-11.06 minutes) and 5.64 fewer minutes of sleep duration (95% CI, 3.98-7.29 minutes) on workdays.

**Table 4.** Associations Between Electronic Screen Use Before Bed and Sleep Duration and Timing in the Cancer Prevention Study–3 Based on Self-Reported Chronotype

Screen use	Chronotype									
	Morning		More morning		None		More evening		Evening	
	Change, mean (95% CI), min	E-value	Change, mean (95% CI), min	E-value	Change, mean (95% CI), min	E-value	Change, mean (95% CI), min	E-value	Change, mean (95% CI), min	E-value
Workday sleep duration^a^										
No.	30 590	NA	30 074	NA	6065	NA	23 341	NA	14 406	NA
No screen use	0 [Reference]	NA	0 [Reference]	NA	0 [Reference]	NA	0 [Reference]	NA	0 [Reference]	NA
Use 1-3 d/wk	−3.17 (−5.09 to −1.26)	1.30	−5.15 (−7.14 to −3.15)	1.41	−0.23 (−5.36 to 4.90)	1.00	−2.84 (−5.48 to −0.20)	1.27	−3.31 (−7.45 to 0.83)	1.00
Use 4-6 d/wk	−5.87 (−7.68 to −4.07)	1.44	−7.48 (−9.35 to −5.61)	1.54	−2.77 (−7.56 to 2.02)	1.00	−7.11 (−9.52 to −4.69)	1.50	−5.46 (−9.19 to −1.74)	1.39
Daily use	−5.64 (−7.29 to −3.98)	1.43	−7.53 (−9.27 to −5.78)	1.54	−6.26 (−10.63 to −1.89)	1.43	−8.20 (−10.47 to −5.93)	1.55	−8.36 (−11.78 to −4.94)	1.53
Workday bedtime^a^										
No.	30 590	NA	30 074	NA	6065	NA	23 341	NA	14 406	NA
No screen use	0 [Reference]	NA	0 [Reference]	NA	0 [Reference]	NA	0 [Reference]	NA	0 [Reference]	NA
Use 1-3 d/wk	4.79 (2.79 to 6.79)	1.38	4.90 (2.91 to 6.89)	1.40	4.84 (−0.64 to 10.31)	1.00	3.56 (0.88 to 6.24)	1.31	0.68 (−3.79 to 5.15)	1.00
Use 4-6 d/wk	9.56 (7.67 to 11.44)	1.61	9.57 (7.71 to 11.44)	1.64	5.07 (−0.05 to 10.18)	1.00	7.71 (5.25 to 10.16)	1.52	5.80 (1.78 to 9.83)	1.39
Daily use	9.33 (7.61 to 11.06)	1.60	9.17 (7.43 to 10.91)	1.62	11.54 (6.88 to 16.21)	1.64	12.71 (10.4 to 15.01)	1.76	15.62 (11.93 to 19.31)	1.79
Nonworkday sleep duration^a^										
No.	35 666	NA	34 832	NA	7234	NA	26 966	NA	16 819	NA
No screen use	0 [Reference]	NA	0 [Reference]	NA	0 [Reference]	NA	0 [Reference]	NA	0 [Reference]	NA
Use 1-3 d/wk	−4.66 (−6.59 to −2.73)	1.36	−6.13 (−8.16 to −4.10)	1.43	0.26 (−5 to 5.53)	1.00	−5.35 (−8.07 to −2.63)	1.37	−6.78 (−11.05 to −2.51)	1.41
Use 4-6 d/wk	−5.58 (−7.41 to −3.75)	1.40	−6.96 (−8.87 to −5.05)	1.47	−3.43 (−8.34 to 1.49)	1.00	−8.21 (−10.7 to −5.71)	1.50	−7.92 (−11.76 to −4.08)	1.46
Daily use	−6.32 (−7.98 to −4.66)	1.44	−6.29 (−8.07 to −4.52)	1.44	−4.02 (−8.47 to 0.43)	1.00	−7.62 (−9.95 to −5.29)	1.48	−10.68 (−14.18 to −7.18)	1.56
Nonworkday bedtime^a^										
No.	35 666	NA	34 832	NA	7234	NA	26 966	NA	16 819	NA
No screen use	0 [Reference]	NA	0 [Reference]	NA	0 [Reference]	NA	0 [Reference]	NA	0 [Reference]	NA
Use 1-3 d/wk	7.06 (4.95 to 9.17)	1.44	5.48 (3.44 to 7.52)	1.40	5.97 (1.00 to 10.94)	1.40	4.18 (1.57 to 6.80)	1.33	3.30 (−1.11 to 7.70)	1.00
Use 4-6 d/wk	10.93 (8.93 to 12.93)	1.61	10.11 (8.19 to 12.03)	1.62	11.05 (6.41 to 15.69)	1.62	8.04 (5.64 to 10.45)	1.51	4.82 (0.86 to 8.78)	1.32
Daily use	9.22 (7.41 to 11.03)	1.54	9.13 (7.34 to 10.91)	1.57	12.10 (7.90 to 16.3)	1.66	12.02 (9.78 to 14.27)	1.69	16.03 (12.42 to 19.64)	1.75

Abbreviation: NA, not applicable.

^a^ Adjusted for age, sex, race, ethnicity, education, bedroom light environment, and sleep mask use; nonworkday models additionally adjusted for work status.

## Discussion

In this large cross-sectional study of US adults, daily electronic screen use prior to sleep was associated with 48 fewer minutes of sleep each week and a 33% higher prevalence of poor sleep compared with those who reported no screen use. The associations between screen use and sleep appeared to differ on the basis of chronotype, with more disruptions to sleep occurring in those reporting a later chronotype. Chronotype is our innate inclination to be active earlier or later in the day. There was a pattern of increasingly delayed bedtimes among daily screen users going from morning to evening chronotypes.

Our results are consistent with those of smaller studies^30,31,32,33,34,35,36^ that have been conducted evaluating electronic screen use before bed in adult populations. In a study of 1225 Australian adults aged 18 and older,^30^ 27% reported daily electronic screen use with a corresponding 21 minutes later weekday and 40 minutes later weekend bedtime. Screen use was associated with later bedtimes, shorter sleep duration, and increased daytime sleepiness among 4188 participants in the Switzerland-based Specchio cohort (aged 19-94 years; 85% women).^37^ Regular electronic screen use in a study of 10 106 Saudi Arabian adults (mean [SD] age, 30.7 [11.3] years) was associated with worse sleep quality (odds ratio range, 1.32-2.12).^31^ Christensen et al^32^ were able to use a mobile application to record objective screen time and reported that screen time before sleep was associated with poorer sleep quality, but not sleep duration. Among a pair of studies in older adults, in a study of 1784 Chinese adults aged 60 to 88 years, 13% reported frequent (almost every day) electronic screen use before bed, which was associated with 45% higher odds of reporting poor sleep (*P* = .03),^34^ whereas in a study of 273 US adults (mean [SD] age, 74.0 [6.5] years),^33^ screen use was associated with 26 fewer minutes of sleep and increased sleep disturbances. Finally, a study of Flemish adults observed poorer sleep and increased fatigue and insomnia among participants with more regular use of a mobile telephone before bed, and shorter sleep durations among their older participants (aged ≥66.36 years).^36^

On average, an individual’s circadian rhythm is slightly longer than 24 hours.^38^ Light exposure is the strongest zeitgeber, the environmental cue that entrains our circadian rhythms to a 24-hour light-dark cycle. Light exposure at night can disrupt sleep by disrupting this natural cycle through delaying the onset of melatonin.^39^ This can lead to reduced sleepiness and increased alertness, which may be reflected by self-reported lower quality of sleep. The increase in ALAN in our environment, coupled with a transition to our more urban lifestyles where we are not exposed to adequate levels of melanopic light (the specific intensity and wavelengths of light that act as a zeitgeber) are factors that are suspected to have contributed to decreased sleep duration the past several decades.^7^ Light therapy, the use of devices that emit melanopic light, has been used for treatment of some sleep phase disorders,^40^ as well as seasonal affective disorder.^41^

Chronotype is latest during adolescence and early adulthood and shifts earlier as we age.^19^ The smaller association between disruptions to sleep and screen use among participants with early chronotypes compared with those with late chronotypes may be due to the more advanced sleep phase among those with early chronotypes countering the external stimuli of screen use; they are naturally more awake and alert in the mornings and drowsy at night.^42,43^ We observed this in our study with those reporting the latest chronotypes having bedtimes over an hour later than those with early chronotypes (eTable in Supplement 1). Those with a later chronotype (35.9% of our population) are at increased risk for poor sleep due social jetlag. Social jetlag is a term that was coined to describe the desynchrony between preferred sleep schedules and sleep dictated by social schedules.^44^ This is most common among those with a later chronotype, and corresponding later bedtimes, needing to awaken earlier than their preference because of work and school start times. This can lead to the accumulation of sleep debt, for which they may compensate by increasing sleep on free days. Exposure to screens, and the corresponding disruption to sleep patterns, may be further exacerbating these effects.

The association between electronic screen use and sleep quality in our adult participants was similar to that reported in a meta-analysis of 20 studies in children and adolescents aged 6 to 19 years old.^16^ Among the 5 studies that assessed sleep quality, the pooled odds ratio was 1.46 (95% CI, 1.14-1.88) for poor sleep. Disruptions to sleep duration were more prominent among children and adolescents than adults. Among the 7 studies in the meta-analysis that evaluated sleep duration, the odds ratio for not meeting recommended sleep duration associated with screen use was 2.17 (95% CI, 1.42-3.32),^16^ whereas a study of fourth and seventh grade students reported 20.6 fewer minutes of sleep for those who slept with a mobile device near their bed.^45^

Disruptions to sleep due to screen use may not be limited to effects of screen light. The National Sleep Foundation recently convened an expert panel to assess the associations between screen use and sleep and was unable to arrive at a consensus for screen light as a factor for sleep disruptions due to screen use.^46^ Consensus voting was on a 9-point scale and required agreement between at least 80% of the panel to vote within a 3-point range. There was agreement that evidence suggests that disruptions to sleep among children and adolescents may be due to psychological factors related to the content being consumed, but no consensus was reached for screen light (75% consensus). However, for adults, there was no consensus on light exposure (56%), content (63%), or the overall association between screen use and sleep (75%). Device manufacturers have been cognizant of possible impacts of blue light exposure prior to sleep and have implemented filters in attempts to reduce such effects, although the efficacy of such filters is still unclear.^12,47,48^ It is not only the light being emitted from these devices that needs to be considered, but the content as well.

Social media is one of the major sources of content being consumed on mobile devices. Although some broader studies have reported on increased sleep disturbances associated with any use of social media,^49,50^ only a handful of studies have looked at social media use at bedtime. A study by Bhat et al^35^ evaluated electronic social media use in bed among hospital employees and university students and observed higher insomnia anxiety and shorter sleep duration among users compared with nonusers. Among adolescents, those who checked social media 30 minutes before bed were 1.62 times more likely to report sleep disturbances.^51^ A large New Zealand study of 4192 adolescents by Galland et al^52^ observed varying reductions to sleep duration based on different activities, ranging from 11 minutes for watching videos to 22 minutes for web browsing. Social media use was associated with a 12-minute reduction, whereas texting was the one behavior for which they did not see a significant association.^52^ However, a recent study of 101 undergraduate students reported no association between bedtime social media use and sleep,^53^ and a sleep laboratory study reported similar findings.^54^ Similar to mobile device uptake patterns, social media use has grown exponentially in adult populations, and additional research is needed into the content they consume and the potential effects on sleep, because it differs greatly from adolescents.^55^

### Strengths and Limitations

Strengths of our study include being the largest to date to evaluate electronic screen use prior to sleeping and sleep outcomes among an adult population. We were able to assess multiple dimensions of sleep, including duration, timing, and quality, and were able to account for the bedroom lighting environment. Although our sleep outcomes were self-reported, we have validated our measure of sleep duration against both sleep diary and actigraphy,^21^ and we used several other well-validated tools.^23,25,26^ We were also able to stratify our results by chronotype to investigate the association between screen use and the internal circadian clock. During our sensitivity analysis, we observed higher E-values among daily users compared with never users, suggesting that any unmeasured confounders would need to exert a stronger effect on the more frequent device users in order to influence the association between device use and sleep.

Limitations of our study include the cross-sectional nature of our assessment of sleep and screen use. This single assessment limited our ability to elucidate the complex day-to-day relationships between other behaviors such as physical activity, diet, and sleep with screen use and social media.^56,57,58^ We do not have additional information beyond weekly frequency of screen use but did observe a trend with increasing days of use reported. Our question regarding screen light was focused on handheld electronic screens and not other light-emitting devices such as televisions or computers. Light intensity reduces exponentially as we move further from the source, so these exposures may be less relevant for potential light-induced sleep disruptions. Results have been mixed, but increasing time spent using these devices has been associated with worse sleep outcomes^33,59,60,61,62^ and may be important to consider as well when evaluating the impact of content on sleep. We also did not have information on other aspects of sleep that may be affected by screen use, such as sleep latency.^63^ Furthermore, our sleep and wake time question was focused on work and nonworkdays. Although we did adjust for self-reported number of workdays, we were unable to evaluate aspects of sleep surrounding retirement or other nonworking statuses. Hagen et al^64^ previous reported differences in weekday sleep duration and timing associated with the retirement transition but found minimal changes to weekend sleep.

## Conclusions

Our findings strengthen the evidence that electronic screen use and disruptions to sleep duration and quality are not limited to children and adolescents but to the broader adult population as well. The decrease in quality and duration appeared to be greater among those with a later chronotype and may be due to delayed bedtimes. Continued work is needed to understand the mechanisms though which screen use disturbs sleep (eg, artificial light at night vs content), especially among individuals with later chronotypes who are already at increased risk of poor sleep due to work and social commitments necessitating earlier wake times.

## Notes

Supplement 1. **eTable**. Association between self-reported chronotype and sleep duration and bedtimes among participants in the Cancer Prevention Study-3 **eFigure 1.** Sleep duration assessment from the 2018 Cancer Prevention Study - 3 questionnaire **eFigure 2.** Participant flow chart

Supplement 2.
Data Sharing Statement
