# Supplementary material for: Electronic Screen Use and Sleep Duration and Timing in Adults
Source: JAMA Netw Open. 2025 Mar 27;8(3):e252493. doi: 10.1001/jamanetworkopen.2025.2493 (PMC11950897; doi:10.1001/jamanetworkopen.2025.2493)
Supplement: Supplement 1. — eTable. Association between self-reported chronotype and sleep duration and bedtimes among participants in the Cancer Prevention Study-3 eFigure 1. Sleep duration assessment from the 2018 Cancer Prevention Study - 3 questionnaire eFigure 2. Participant flow chart [file jamanetwopen-e252493-s001.pdf]

Supplemental Online Content

Zhong C, Masters M, Donzella SM, Diver WR, Patel AV. Electronic screen use and sleep duration and timing in adults. *JAMA Netw Open*. 2025;8(3):e252493. doi:10.1001/jamanetworkopen.2025.2493

eTable. Association between self-reported chronotype and sleep duration and bedtimes among participants in the Cancer Prevention Study-3

eFigure 1. Sleep duration assessment from the 2018 Cancer Prevention Study - 3 questionnaire

eFigure 2. Participant flow chart

This supplemental material has been provided by the authors to give readers additional information about their work.

© 2025 Zhong C et al. *JAMA Network Open*.

eTable. Association between self-reported chronotype and sleep duration and bedtimes among participants in the Cancer Prevention Study-3

	Workday (N=104,476)				Non-Workday (N=121,517)			
	Duration (minutes)	e-value	Bedtime (minutes)	e-value	Duration (Minutes)	e-value	Bedtime (Minutes)	e-value
Morning	-3.31 (-4.82, -1.81)	1.30	-34.4 (-35.96, -32.84)	2.69	-13.03 (-14.59, -11.48)	1.71	-35.01 (-36.6, -33.42)	2.54
More morning	0.70 (-0.81, 2.20)	1.00	-13.75 (-15.31, -12.19)	1.74	-0.95 (-2.51, 0.60)	1.00	-12.65 (-14.24, -11.06)	1.64
None	reference		reference		reference		reference	
More evening	-0.34 (-1.89, 1.20)	1.00	11.15 (9.55, 12.75)	1.63	6.03 (4.43, 7.62)	1.41	15.35 (13.71, 16.98)	1.75
Evening	-11.67 (-13.31, -10.03)	1.72	43.69 (41.99, 45.39)	3.19	2.57 (0.87, 4.26)	1.26	53.05 (51.32, 54.78)	3.43

Adjusted for age, sex, race, ethnicity, education, bedroom light environment, and sleep mask use; non-workday models additionally adjusted for work status

© 2025 Zhong C et al. *JAMA Network Open*.

eFigure 1. Sleep duration assessment from the 2018 Cancer Prevention Study - 3 questionnaire

Over the past year, what time do you typically try to **fall asleep** and **wake up** on WORKDAYS and on NON-WORKDAYS. If you did not work in the past year, leave the WORKDAY boxes blank.

	**Fall Asleep**	**Wake Up**	**Over the past year, on average how many days per week were:**	
WORKDAY	: ☐ am ☐ pm	: ☐ am ☐ pm	WORKDAYS	
NON-WORKDAY	: ☐ am ☐ pm	: ☐ am ☐ pm	NON-WORKDAYS	

© 2025 Zhong C et al. *JAMA Network Open*.

eFigure 2. Participant flow chart

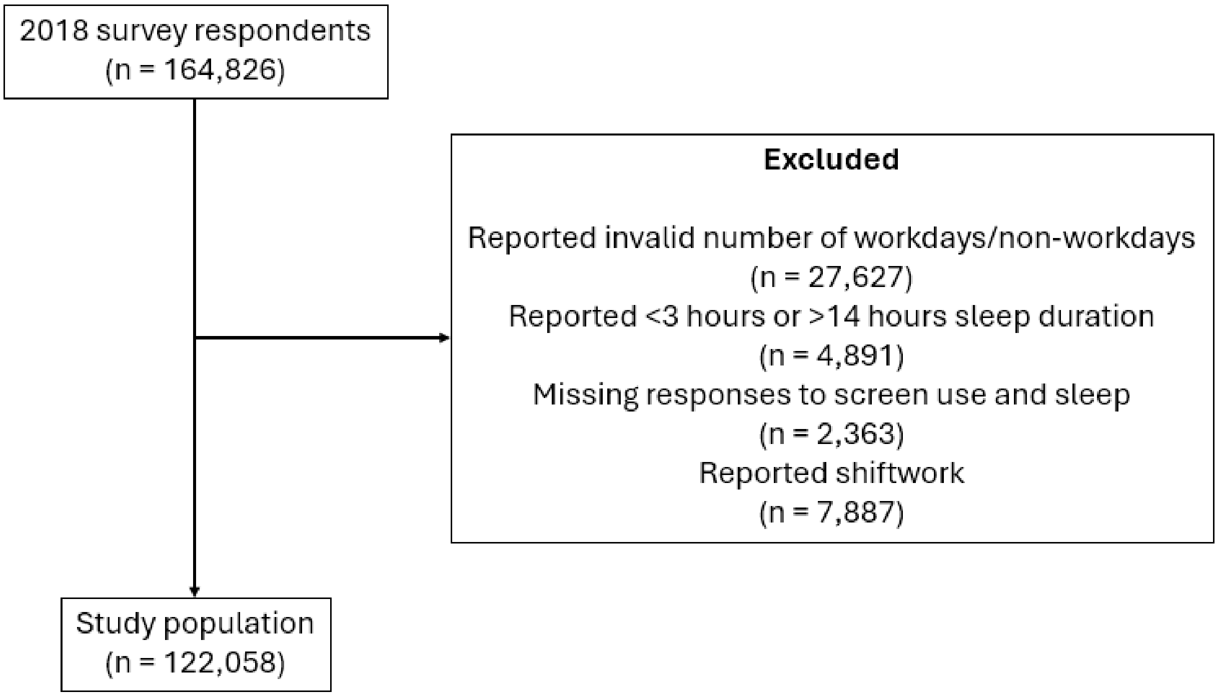

© 2025 Zhong C et al. *JAMA Network Open*.
