# Supplementary material for: Electronic Screen Use and Sleep Duration and Timing in Adults
Source: JAMA Netw Open. 2025 Mar 27;8(3):e252493. doi: 10.1001/jamanetworkopen.2025.2493 (PMC11950897; doi:10.1001/jamanetworkopen.2025.2493)
Supplement: Supplement 2. — Data Sharing Statement [file jamanetwopen-e252493-s002.pdf]

Data Sharing Statement

Zhong. Electronic Screen Use and Sleep Duration and Timing in Adults. *JAMA Netw Open*. Published March 27, 2025. doi:10.1001/jamanetworkopen.2025.2493

Data
Data available: No

Additional Information
Explanation for why data not available: Data are available from the American Cancer Society by following the ACS Data Access Procedures (https://www.cancer.org/content/dam/cancer-org/research/epidemiology/cancer-prevention-study-data-access-policies.pdf[MH1]) for researchers who meet the criteria for access to confidential data. Please email cohort.data@cancer.org to inquire about access.
